# Surface Charge-Dependent Cellular Uptake of Polystyrene Nanoparticles

**DOI:** 10.3390/nano8121028

**Published:** 2018-12-10

**Authors:** Soyeon Jeon, Jessica Clavadetscher, Dong-Keun Lee, Sunay V. Chankeshwara, Mark Bradley, Wan-Seob Cho

**Affiliations:** 1 Lab of Toxicology, Department of Medicinal Biotechnology, College of Health Sciences, Dong-A University, Busan 49315, Korea; wjsthdus0418@naver.com (S.J.); dnjsxo356@naver.com (D.-K.L.); 2 EastChem, School of Chemistry, University of Edinburgh, David Brewster Road, Edinburgh EH9 3FJ, UK; jessica.clavadetscher@gmail.com (J.C.); sunay.chankeshwara@astrazeneca.com (S.V.C.); mark.bradley@ed.ac.uk (M.B.); 3 Medicinal Chemistry, Cardiovascular, Renal and Metabolism, IMED Biotech Unit, AstraZeneca, Pepparedsleden 1, 431 50 Mölndal, Sweden

**Keywords:** cellular uptake, fluorescence, surface charge, polystyrene nanoparticles, macrophage, epithelial cell

## Abstract

The evaluation of the role of physicochemical properties in the toxicity of nanoparticles is important for the understanding of toxicity mechanisms and for controlling the behavior of nanoparticles. The surface charge of nanoparticles is suggested as one of the key parameters which decide their biological impact. In this study, we synthesized fluorophore-conjugated polystyrene nanoparticles (*F*-PLNPs), with seven different types of surface functional groups that were all based on an identical core, to evaluate the role of surface charge in the cellular uptake of nanoparticles. Phagocytic differentiated THP-1 cells or non-phagocytic A549 cells were incubated with *F*-PLNP for 4 h, and their cellular uptake was quantified by fluorescence intensity and confocal microscopy. The amount of internalized *F*-PLNPs showed a good positive correlation with the zeta potential of *F*-PLNPs in both cell lines (Pearson’s *r* = 0.7021 and 0.7852 for zeta potential vs. cellular uptake in THP-1 cells and nonphagocytic A549 cells, respectively). This result implies that surface charge is the major parameter determining cellular uptake efficiency, although other factors such as aggregation/agglomeration, protein corona formation, and compositional elements can also influence the cellular uptake partly or indirectly.

## 1. Introduction

Nanomaterials are applied as various biomedical tools including diagnostic, monitoring, and therapeutic tools [1,2,3,4,5]. However, the surface modification of nanomaterials, which is essential for these applications to improve their physicochemical properties, can also affect the biocompatibility of the materials by changing their inflammogenic potential [6,7], cytotoxicity [8], or toxicokinetics [9]. Because the toxicity outcomes of nanomaterials originate from their physicochemical properties, nanomaterials with different physicochemical properties (e.g., surface charge, shape, size, and surface reactivity) may have different toxicity potentials and toxicological outcomes [10].

Among various physicochemical properties, surface charge is suggested as one of the major factors which control various biological responses to nanomaterials. In the lung inflammation model, the value of zeta potential showed a good positive correlation with the inflammogenic potential of metal-oxide nanoparticles or polystyrene nanoparticles [7,11]. In biodistribution studies, positively charged polyethylene glycol/polymeric nanoparticles showed a favorable distribution and higher bioavailability in Caco-2 cells and in vivo models compared to negatively charged particles [12]. Positively charged magnetite nanoparticles were better internalized in human breast cancer cells than negatively charged ones, but the surface charges did not influence internalization in human umbilical vein endothelial cells [13]. Positively charged particles were shown to have a higher uptake efficiency than negatively charged particles; however, there are still discrepancies between studies and further studies using well-engineered nanoparticles, and relevant cell types are needed.

Inhalation is the major route of nanoparticle exposure and is associated with a relatively higher risk than oral and dermal exposures. The primary target cell of inhaled nanoparticles is the alveolar macrophage, and the secondary target is the alveolar epithelial cell [10,14]. Herein, we synthesized seven different well-engineered, charged polystyrene nanoparticles via conjugation to surface functional groups to investigate the role of surface charge in cellular uptake. Because the main limitation for the quantitative analysis of polystyrene nanoparticles is the difficulty associated with their detection, we co-polymerized a green fluorophore with the nanoparticles and evaluated the effect of surface charge on the levels of cellular uptake using THP-1 macrophages and A549 cells.

## 2. Materials and Methods

### 2.1. Synthesis of Fluorescein-Conjugated Polystyrene Nanoparticles (*F*-PLNPs)

Fluorescein aminomethyl polystyrene nanoparticles (_NH2_*F*-PLNPs) were synthesized as the core nanoparticle, and their surface was modified by conjugating six different functional groups (guanidinium, polyethylene glycol, acetyl, zwitterionic, carboxyl, and sulfonic acid) to provide diverse surface charges while minimizing variability in other factors. The detailed method of *F*-PLNP synthesis was described previously [15,16]. Because each batch of samples can have different concentrations of functional groups and fluorophores, a large batch of samples was prepared to enable the completion of experiments within a single batch of nanoparticles.

### 2.2. Characterization of NPs

The size and morphology of PLNPs were evaluated using transmission electron microscopy (TEM) and scanning electron microscopy (SEM). Briefly, each PLNP sample in distilled water (DW) was loaded onto a copper grid and measured using a TEM (JEOL, Tokyo, Japan). For SEM analysis, 200 μg of nanoparticles in DW was loaded onto carbon-coated stubs and dried overnight under vacuum (<20 bar) at 40 °C. After gold-sputter coating, the particles were visualized with SEM (Phillips, Eindhoven, The Netherlands). The hydrodynamic size of the PLNPs in DW was measured using a Malvern Zetasizer Nano-ZS (Malvern, Malvern Hills, UK). The surface charge of PLNPs was also measured using a Malvern Zetasizer Nano-ZS not only in DW, but also in actual media where PLNPs were in contact with phenol red-free Roswell Park Memorial Institute-1640 (RPMI-1640; Corning, Corning, NY, USA) for THP-1 cells and phenol red-free Dulbecco’s modified Eagle medium (DMEM; Corning) for A549 cells. Because each sample of *F*-PLNP has different fluorescence intensity, the fluorescence intensity of *F*-PLNP was measured using a Becton Dickinson Biosciences FACS Aria (Wokingham Berkshire, UK), and data analysis was performed using FlowJo Version 7.6.3 software (Tree Star, Ashland, OR, USA). The endotoxin level of PLNPs was measured using an endpoint chromogenic limulus amebocyte lysate (LAL) assay kit (Cambrex, Walkersville, MD, USA). The concentration of PLNPs for the LAL assay was 50 µg/mL in DW (the highest dose for in vitro study), and the LAL assay was performed according to the manufacturer’s instructions. The detection limit for this assay is 0.1–1.0 endotoxin units (EU)/mL.

### 2.3. Study Design for Cellular Uptake Experiments

**Figure 1:**
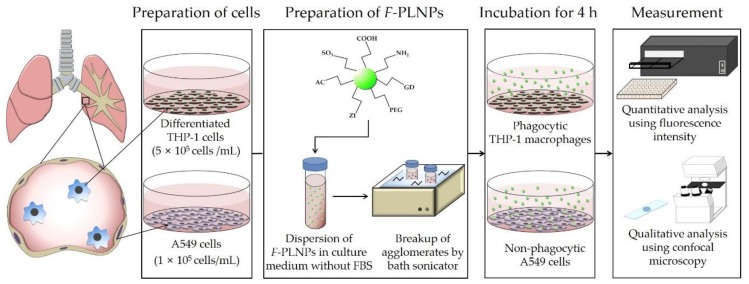
Study design of fluorophore-conjugated polystyrene nanoparticle (*F*-PLNP) uptake in THP-1 cells and A549 cells.

The study design is presented in Figure 1. To evaluate the role of the surface charge of nanoparticles in cellular uptake, macrophage-like THP-1 cells and lung epithelial A549 cells were incubated with seven different PLNPs having different surface functional groups. Because the addition of fetal bovine serum (FBS) can mask the surface of nanoparticles due to protein corona formation [6], FBS was excluded from the nanoparticle treatment. Before being applied to the cells, nanoparticles were dispersed in culture medium and sonicated for 10 min in a bath sonicator (Saehan-Sonic, Seoul, Korea) to break up agglomerations. Then, cells were treated with the nanoparticle suspensions for 4 h. The selection of 4-h incubation was decided based on two reasons. Firstly, the Organization for Economic Co-operation and Development (OECD) TG 403 (acute inhalation toxicity) suggested the duration of inhalation exposure to be up to 6 h in rats and up to 4 h in mice. Therefore, a 4-h incubation period in this study can reflect the acute inhalation study. Secondly, this study did not evaluate cytotoxic potential, but rather, cellular uptake efficiency. Shoshi et al. [17] showed that 4-h incubation is about the steepest slope for the cellular uptake kinetics of nanoparticles, and 24-h incubation can saturate cells, thereby obstructing the differential cellular uptake efficiency due to surface modifications. Also, dos Santos et al. [18] showed that the uptake kinetics of nanoparticles in various cells, including macrophages (RAW 264.7) and lung epithelial cells (A549), was almost linear up to 4 h. Our previous study also showed that polystyrene nanoparticles could induce cytotoxicity after 4-h incubation with nanoparticles and a further 20-h cell culture followed by washing unbound nanoparticles from cells [8]. Therefore, we decided that the 4-h incubation was an appropriate time point to evaluate the cellular uptake efficiency of nanoparticles.

### 2.4. Cellular Uptake Assay Using Macrophage-Like THP-1 Cells

Monocytic THP-1 cells were purchased from Korean Cell Line Bank (Seoul, Korea). THP-1 cells were cultured in RPMI-1640 (Corning) medium containing 10% FBS (Corning), 2 mM l-glutamine (Life Technologies, Gaithersburg, MD, USA), 100 international units (IU)/mL penicillin (Life Technologies), and 100 U/mL streptomycin (Life Technologies). Monocytic THP-1 cells were triggered to differentiate into macrophage-like cells by treatment with 10 ng/mL of phorbol myristate acetate (PMA; Sigma-Aldrich, St Louis, MO, USA) for two days before the experiments. Cells were cultured at 37 °C in a 5% CO_2_ humidified environment. The levels of cellular uptake of PLNPs by macrophage-like THP-1 cells were measured using the fluorescence intensity, and cellular uptake was confirmed using confocal microscopy. Briefly, differentiated THP-1 cells at a density of 5 × 10^5^ cells/mL were cultured in black 96-well plates (Corning). Before treatment of PLNPs, cells were cultured with RPMI-1640 medium containing 10% FBS. PLNPs were dispersed in phenol red-free RPMI-1640 medium (Corning) without FBS supplementation to avoid the fluorescence interference by phenol red and the masking of the surface charge of PLNPs by protein corona formation. PLNPs were added at 50 μg/mL and the cells were incubated for 4 h. After incubation, a total of 150 μL of supernatant within each well was collected after simple agitation, and the remaining medium was washed three times with pre-warmed phosphate-buffered saline. Then, fresh phenol red-free RPMI-1640 medium was added. The fluorescence intensity of each sample was measured using a microplate reader (Synergy HT, BioTek, Seoul, Korea) at 490/528 nm. To confirm the cellular uptake, cells were attached to glass slides after cytospin preparation (4 × 10^4^ cells/slide) followed by treatment with Accutase (Sigma-Aldrich). The slides were stained with 4′,6-diamidino-2-phenylindole (DAPI; Abcam, Cambridge, UK) and observed under a confocal laser scanning microscope (CLSM) (LSM 700: ZEISS, Oberkochen, Germany) to image the cellular uptake of *F*-PLNPs.

### 2.5. Cellular Uptake Assay Using Lung Epithelial A549 Cells

A549 cells were purchased from Korean Cell Line Bank and cultured in DMEM (Corning) supplemented with 5% FBS, 2 mM l-glutamine, 100 IU/mL penicillin, and 100 U/mL streptomycin. A549 cells were seeded at 1 × 10^5^ cells/mL on a black 96-well plate and cultured overnight. Then *F*-PLNPs were dispersed at 50 μg/mL in phenol red-free DMEM medium without FBS and the cells were treated for 4 h. The preparation and fluorescence measurement of samples were performed with the same methods described for THP-1 cells except 0.05% trypsin/ethylenediaminetetraacetic acid (EDTA) (Life Technologies) instead of Accutase was used to detach cells.

### 2.6. Statistical Analysis

Data are presented as the mean ± standard error of mean, and were analyzed using GraphPad Prism Software (version 6; GraphPad Software Inc., La Jolla, CA, USA). Pearson’s correlation test was applied to determine the correlation between zeta potentials of PLNP and levels of cellular uptake.

## 3. Results and Discussion

### 3.1. Physicochemical Properties of *F*-PLNPs

**Figure 2:**
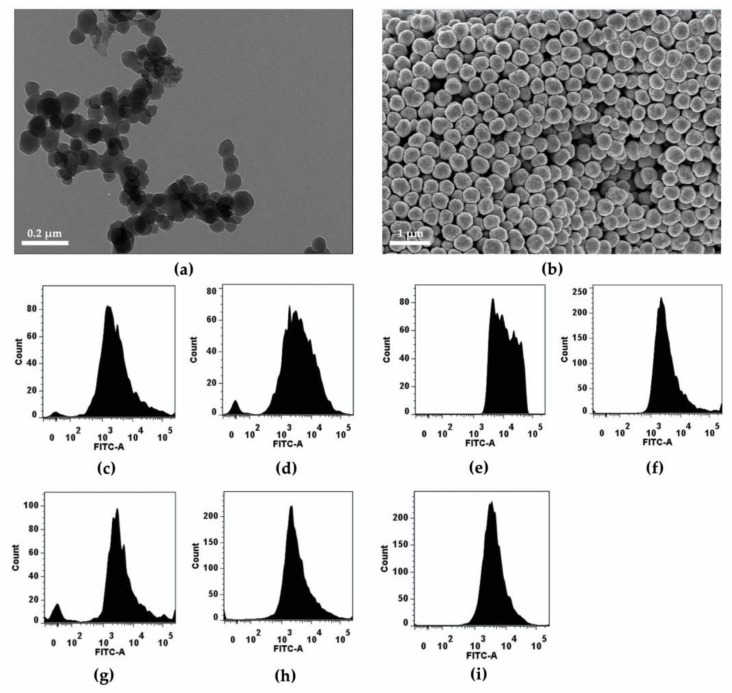
Morphology and fluorescence intensity of nanoparticles. The representative (**a**) TEM and (**b**) SEM image of amine (_NH2_)*F*-PLNPs. Fluorescence intensity (**c**–**i**) of nanoparticles was analyzed using flow cytometry: (**c**) (_NH2_)*F*-PLNP, (**d**) guanidium (_GD_)*F*-PLNP, (**e**) polyethylene glycol (_PEG_)*F*-PLNP, (**f**) acetyl (_AC_)*F*-PLNP, (**g**) zwitterionic (_ZW_)*F*-PLNP, (**h**) carboxyl (_COOH_)*F*-PLNP, and (**i**) sulfonic (_SO3_)*F*-PLNP.

Fluorescein-polymerized aminomethyl polystyrene nanoparticles, the core nanoparticles, were synthesized and further modified by conjugating six different functional groups (guanidinium, polyethylene glycol, acetyl, zwitterionic, carboxyl, and sulfonic acid) to provide diverse surface charges while minimizing the variability of other factors. Seven different fluorescein-containing polystyrene nanoparticles were synthesized: amine (_NH2_*F*-PLNP), guanidinium (_GD_*F*-PLNP), polyethylene glycol (_PEG_*F*-PLNP), zwitterionic (_ZW_*F*-PLNP), acetyl (_AC_*F*-PLNP), carboxyl (_COOH_*F*-PLNP), and sulfonic acid (_SO3_*F*-PLNP). The primary size and shape of nanoparticles, as shown by SEM and TEM, revealed a uniform spherical shape with a diameter of 82.6 ± 1.2 nm (Figure 2a,b). Each type of nanoparticle expressed good fluorescence intensity in the fluorescein isothiocyanate (FITC) channel, with some variability between nanoparticles (Figure 2c–i). The physicochemical properties are summarized in Table 1. Although the primary size was identical for all nanoparticles, the hydrodynamic size varied between 254 and 429 nm. Among nanoparticles, _COOH_*F*-PLNP showed better dispersion, whereas _GD_*F*-PLNP showed worse dispersion compared to the others. The polydispersity index of nanoparticles was less than 0.3, which implies that all types of nanoparticles had a narrow size distribution. The zeta potential of nanoparticles ranged from −41 to +42 mV in DW, while the zeta potential of nanoparticles in culture media showed negative charges for all nanoparticles without overlapping between nanoparticles: −42 to −27 mV in phenol red-free RPMI-1640 and −47 to −32 mV in phenol red-free DMEM. All types of nanoparticles showed no endotoxin contamination at 50 μg/mL (which is the highest dose for in vitro studies).

### 3.2. Cytotoxicity Assay of Nanoparticles Against THP-1 Macrophages and A549 Cells

**Figure 3:**
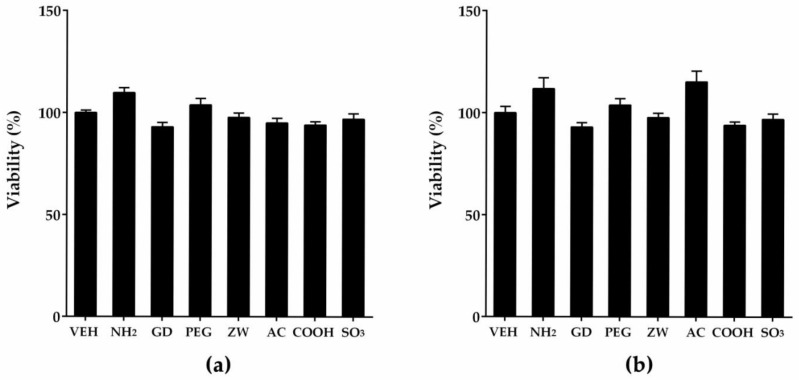
Cytotoxicity assay of nanoparticles to (**a**) THP-1 macrophages and (**b**) A549 cells. Cells were treated with nanoparticles at 50 μg/mL for 4 h, and cytotoxicity was evaluated using the 3-(4,5-dimethylthiazol-2-yl)-5-(3-carboxymethoxyphenyl)-2-(4-sulfophenyl)-2H-tetrazolium (MTS) assay. Note that all types of nanoparticles showed no cytotoxicity at 50 μg/mL. VEH—vehicle control; NH_2_—amine; GD—guanidium; PEG—polyethylene glycol; ZW—zwitterionic; AC—acetyl; COOH—carboxyl; SO_3_—sulfonic.

To evaluate the role of surface charge or surface modification on the cellular uptake, cell lines were selected based on their relevance to the inhalation setting. Because the primary and secondary targets for inhaled particles are alveolar macrophages and alveolar epithelial cells, respectively, differentiated THP-1 cells and A549 cells were selected as relevant cell lines. To perform experiments of the comparative cellular uptake of nanoparticles, the selection of dose is critical, because excessively high dose levels cannot show differential cellular uptake due to particle overload and/or cell death by nanoparticles. Likewise, dose levels that are too low might not provide measurable fluorescence intensities. Therefore, we performed cytotoxicity assays of nanoparticles on THP-1 macrophages and A549 cells, and found that 50 μg/mL was the dose at which differential cellular uptake occurred without cytotoxicity in both cell lines (Figure 3). Although A549 cells could be treated with a higher dose of nanoparticles, identical doses were applied to both cell types because a higher dosage can produce particle overload and saturation of cellular uptake. In our previous study, 24-h treatment of 50 μg/mL of non-fluorescent PLNPs to both THP-1 macrophages and A549 cells showed less than 5% cytotoxicity, while 24-h treatment of 100 μg/mL of certain types of PLNPs showed significant cytotoxicity to both cell lines [8]. However, 4-h treatments showed no cytotoxicity in both cell lines even at 100 μg/mL [8]. Another study showed that carboxylated and aminated PLNPs added at 50 μg/mL for 38 h were not cytotoxic to primary cultured human macrophages, THP-1 monocytes, and THP-1 macrophages [19].

### 3.3. Zeta Potential-Dependent Cellular Uptake of Nanoparticles in Phagocytic THP-1 Macrophages

**Figure 4:**
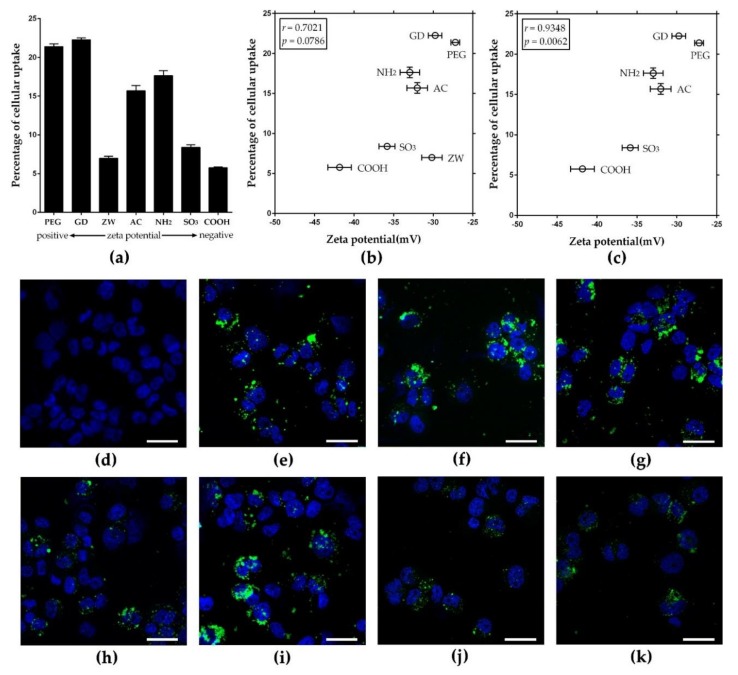
Cellular uptake of *F*-PLNPs at 50 μg/mL by THP-1 macrophages. (**a**) The percentage of cellular uptake of *F*-PLNPs evaluated by fluorescence intensity. (**b**) Plotting the values of zeta potential in Roswell Park Memorial Institute medium (RPMI-1640) against percentages of cellular uptake, yielding a Pearson’s correlation coefficient of 0.7021. (**c**) Correlation plot upon excluding _Z__W_*F*-PLNP of Figure 4b showed a much improved Pearson’s correlation coefficient (0.9348, *p* = 0.0062). Data are presented as means ± standard error of the mean (SEM); *n* = 4. Confocal laser scanning microscopy (CLSM) images of (**d**) vehicle control, (**e**) amine (_NH2_)*F*-PLNP, (**f**) guanidium (_GD_)*F*-PLNP, (**g**) polyethylene glycol (_PEG_)*F*-PLNP, (**h**) zwitterionic (_ZW_)*F*-PLNP, (**i**) acetyl (_AC_)*F*-PLNP, (**j**) carboxyl (_COOH_)*F*-PLNP, and (**k**) sulfonic (_SO3_)*F*-PLNP. Blue fluorescence, 4′,6-diamidino-2-phenylindole (DAPI) staining; green fluorescence, *F*-PLNPs. Scale bar: 30 μm.

To evaluate the role of surface charge on the cellular uptake by phagocytic cells, seven types of nanoparticles were added to THP-1 macrophages at 50 μg/mL for 4 h, and intracellular fluorescence intensity was measured after washing the cells to remove the extracellular nanoparticles. With the initial treated dose, three out of four positively charged PLNPs showed about 15–21% cellular uptake, while two out three negatively charged PLNPs showed about 5–8% cellular uptake (Figure 4a). The correlation plot between zeta potentials in RPMI-1640 and percentages of cellular uptake showed an excellent correlation (Pearson’s correlation coefficient: 0.7021) (Figure 4b). The Pearson’s correlation coefficient could be adjusted to 0.9348 (*p* = 0.0062) if _Z__W_*F*-PLNP was considered as an outlier (Figure 4c). Previous studies show that zwitterionic polymers could resist protein adsorption [20,21], which can provide a stealth effect and reduce cellular uptake of nanoparticles by phagocytes although they may be positively charged [22,23]. In addition, the correlation coefficient between the zeta potential in DW and the percentage of cellular uptake was 0.6706 (Figure S1a, b, Supplementary Materials). The cellular uptake was confirmed by imaging intracellular PLNPs using CLSM (Figure 4d–k). CLSM images showed that PLNPs were located in the cytoplasm, and the amount of phagocytosed PLNPs was consistent with the correlation data. This result implies that positively charge NPs were taken up by THP-1 macrophages at a higher rate than negatively charged ones. In our previous study, treating THP-1 macrophages with non-fluorescent PLNPs, the zeta potential of PLNPs correlated positively with cytotoxicity; moreover, the concentration of coronated protein on the surface of PLNPs showed a negative correlation with cytotoxicity [8]. Intratracheal instillation of *F*-PLNPs to rats in our previous study also showed that the zeta potential of *F*-PLNPs correlated positively with the inflammogenic potential of *F*-PLNPs, and the mechanism of inflammation was identical regardless of surface functionalization [7]. Therefore, the result of this study implies that the greater uptake of nanoparticles due to the higher zeta potentials can provide increased dosage to cells without changing the mechanism of toxicity. The positive correlation between zeta potential and cellular uptake found in this study using seven types of PLNPs was consistent with several previous studies using small sets of nanoparticles in various cell types including HeLa cells [24], NR8383 [25], Caco-2 cells [12], and macrophages [26]. In addition, the uptake mechanism of nanoparticles can be different, depending on the types of nanoparticles, as a previous study showed that positively charged PLNPs were internalized via clathrin receptors, while negatively charged PLNPs were internalized via caveolin receptors. The difference in the mechanism of uptake can also influence the amount of cellular uptake [25]. The correlation plot between hydrodynamic size and cellular uptake efficacy in THP-1 macrophages showed no direct correlation between these two parameters (Figure S2a, Supplementary Materials). This result is consistent with the previous studies which suggested that agglomeration size did not reflect the cytotoxicity and dissolution efficacy, but that the primary size is more important than the size of the agglomerates for silver nanoparticles [27,28,29]. However, regarding cellular uptake, the hydrodynamic size of the nanoparticle can partly or indirectly influence the cellular uptake efficacy [30,31,32]. In our recent study, we suggested the dual contribution of surface charge and protein-binding affinity to the cytotoxicity of PLNPs [8].

### 3.4. Zeta Potential-Dependent Cellular Uptake of Nanoparticles in Non-Phagocytic A549 Cells

**Figure 5:**
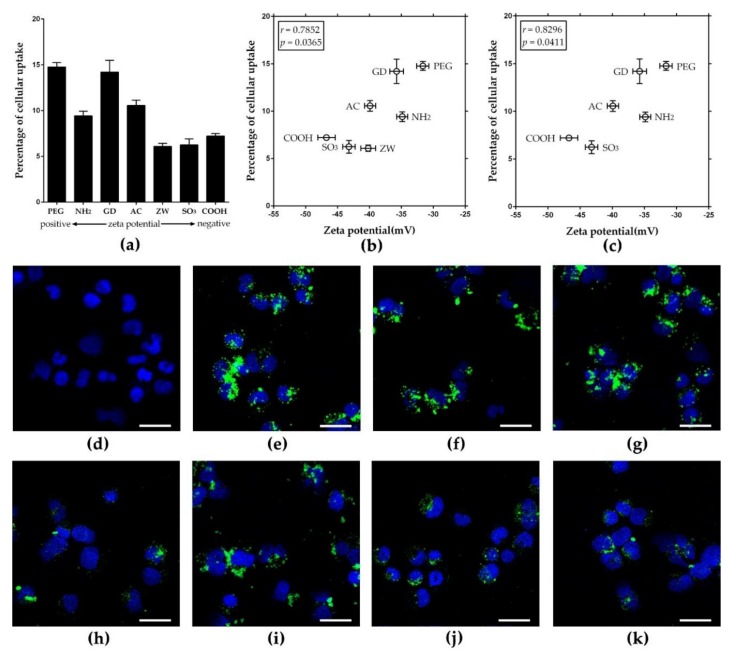
Cellular uptake of *F*-PLNPs at 50 μg/mL by the non-phagocytic A549 cells. (**a**) The percentage of cellular uptake of *F*-PLNPs determined by fluorescence intensity. (**b**) Plotting the values of zeta potential in Dulbecco’s modified Eagle medium (DMEM) against percentages of cellular uptake, yielding the Pearson’s correlation coefficient of 0.7852. (**c**) Correlation plot upon excluding (_Z__W_)*F*-PLNP of Figure 5b showed a much improved Pearson’s correlation coefficient (0.8296, *p* = 0.0411). Data are presented as means ± SEM; *n* = 4. CLSM images of (**d**) vehicle control, (**e**) amine (_NH2_)*F*-PLNP, (**f**) guanidium (_GD_)*F*-PLNP, (**g**) polyethylene glycol (_PEG_)*F*-PLNP, (**h**) zwitterionic (_ZW_)*F*-PLNP, (**i**) acetyl (_AC_)*F*-PLNP, (**j**) carboxyl (_COOH_)*F*-PLNP, and (**k**) sulfonic (_SO3_)*F*-PLNP. Blue fluorescence, DAPI staining; green fluorescence, *F*-PLNPs. Scale bar: 30 μm.

To evaluate the role of surface charge in the cellular uptake in non-phagocytic cells, seven types of nanoparticles were added to A549 cells at 50 μg/mL for 4 h, and intracellular fluorescence intensity was measured after washing cells to remove extracellular nanoparticles. With the initial treated dose, three out of four positively charged nanoparticles showed about 10–15% cellular uptake, while two out of three negatively charged nanoparticles showed approximately 6–7% cellular uptake (Figure 5a). The pattern of cellular uptake was similar to that of THP-1 macrophages, although THP-1 macrophages had higher amounts of cellular uptake than A549 cells. The data are consistent with previous observations in THP-1 macrophages, apart from the _Z__W_*F*-PLNP uptake, which might be due to the stealth effect of zwitterionic polymers [22,23]. The correlation plot between the value of zeta potential and percentage of cellular uptake showed a good positive correlation (Pearson’s correlation coefficient: 0.7852) (Figure 5b). The Pearson’s correlation coefficient could be adjusted to 0.8296 (*p* = 0.0411) if _Z__W_*F*-PLNP was considered as an outlier (Figure 5c). In addition, the correlation coefficient between the zeta potential in DW and the percentages of cellular uptake was 0.5523 (Figure S1c, d, Supplementary Materials).

This result implies that a higher zeta potential is associated with more cellular uptake on nanoparticles, which is consistent with the observations in THP-1 macrophages. The intracellular localization of nanoparticles was confirmed using CLSM images (Figure 5d–k). In addition to surface charge, several physicochemical factors such as size, agglomeration status, and shape can influence the cellular uptake of nanoparticles [33,34]. In this study, we minimized these other factors by conjugating various functional groups to identical monodispersed PLNPs. The correlation plot between hydrodynamic size and cellular uptake efficacy in A549 cells showed no direct correlation between these two parameters, which is consistent with the data shown in THP-1 macrophages (Figure S2b, Supplementary Materials). Therefore, the results of this study imply that surface charge is a major factor determining the cellular uptake of PLNPs, and the intrinsic properties of conjugated surface functional groups can have additional effects such as the stealth effect shown by _Z__W_*F*-PLNPs.

## 4. Conclusions

The seven different types of *F*-PLNPs with identical core showed different cellular uptake efficacies in both phagocytic THP-1 macrophages and non-phagocytic A549 cells. However, there was a positive trend between the zeta potential and the cellular uptake efficacy, and the correlation was clearer in the phagocytic THP-1 macrophages. This result implies that surface charge is the major parameter determining cellular uptake efficiency, although other factors such as aggregation/agglomeration, protein corona formation, and compositional elements can also influence the cellular uptake partly or indirectly.

## Figures and Tables

**Table 1:** Physicochemical properties of the nanoparticles.

PLNPs	Zeta Potential (mV)			Hydrodynamic Size (nm)	Polydispersity Index (PDI)	Endotoxin (EU/mL)
	In DW	In RPMI-1640	In DMEM			
_NH2_*F*-PLNP	42.1 ± 1.0	−33.0 ± 1.3	−34.9 ± 0.9	324.2 ± 4.6	0.3 ± 0.0	Not detected
_GD_F-PLNP	39.7 ± 3.6	−29.8 ± 0.9	−35.8 ± 1.1	429.6 ± 9.6	0.3 ± 0.0	
_PEG_*F*-PLNP	27.3 ± 1.9	−27.2 ± 0.6	−31.7 ± 1.0	280.6 ± 1.4	0.1 ± 0.0	
_ZW_F-PLNP	24.2 ± 1.5	−30.2 ± 1.3	−40.2 ± 1.2	369.1 ± 2.6	0.3 ± 0.0	
_AC_*F*-PLNP	−17.8 ± 1.1	−32.1 ± 1.3	−39.9 ± 0.9	301.1 ± 3.5	0.2 ± 0.0	
_COOH_*F*-PLNP	−33.4 ± 0.8	−41.9 ± 1.5	−46.8 ± 1.4	254.4 ± 0.9	0.0 ± 0.0	
_SO3_*F*-PLNP	−40.9 ± 0.6	−35.9 ± 1.1	−43.3 ± 1.0	258.4 ± 1.4	0.0 ± 0.0	

*F*-PLNP—fluorophore-conjugated polystyrene nanoparticle; NH_2_—amine; GD—guanidium; PEG—polyethylene glycol; ZW—zwitterionic; AC—acetyl; COOH—carboxyl; SO_3_—sulfonic; DW—distilled water; RPMI—Roswell Park Memorial Institute; DMEM—Dulbecco’s modified Eagle medium; EU—endotoxin units.

## Supplementary Materials

The following are available online at http://www.mdpi.com/2079-4991/8/12/1028/s1. Figure S1: Plotting of values of zeta potential in DW against percentages of cellular uptake. The zeta potential of nanoparticles was plotted against percentages of cellular uptake in THP-1 macrophages using all types of nanoparticles (a) or excluding zwitterionic _(ZW)_*F*-PLNP (b) which considered as an outlier. The zeta potential of nanoparticles was plotted against percentages of cellular uptake in A549 cells using all types of nanoparticles (c) or excluding zwitterionic _(ZW)_*F*-PLNP (d) which considered as an outlier. The Pearson’s correlation test was applied. Data are presented as mean ± SEM and *n* = 4; Figure S2: Plotting of values of hydrodynamic size against percentages of cellular uptake. The hydrodynamic size of nanoparticles was plotted against percentages of cellular uptake in THP-1 macrophages (a) and A549 cells (b). The Pearson’s correlation test was applied. Data are presented as mean ± SEM and *n* = 4.
