# Supplementary material for: Surface Charge-Dependent Cellular Uptake of Polystyrene Nanoparticles
Source: Nanomaterials (Basel). 2018 Dec 10;8(12):1028. doi: 10.3390/nano8121028 (PMC6316338; doi:10.3390/nano8121028)
Supplement: Supplementary file 1 [file nanomaterials-08-01028-s001.pdf]

Supplementary File

Surface Charge-Dependent Cellular Uptake of Polystyrene Nanoparticles

Soyeon Jeon [1,†], Jessica Clavadetscher [2,†], Dong-Keun Lee [1], Sunay V. Chankeshwara [2,3], Mark Bradley [2] and Wan-Seob Cho [1,*]

1 Lab of Toxicology, Department of Medicinal Biotechnology, College of Health Sciences, Dong-A University, Busan 49315, Korea; wjsthdus0418@naver.com (S.J.); dnjsxo356@naver.com (D.-K.L.)

2 EastChem, School of Chemistry, University of Edinburgh, David Brewster Road, Edinburgh EH9 3FJ, UK; jessica.clavadetscher@gmail.com (J.C.); sunay.chankeshwara@astrazeneca.com (S.V.C.); mark.bradley@ed.ac.uk (M.B.)

3 Medicinal Chemistry, Cardiovascular, Renal and Metabolism, IMED Biotech Unit, AstraZeneca, Pepparedsleden 1, 431 50 Mölndal, Sweden

* Correspondence: wcho@dau.ac.kr; Tel.: +82-51-200-7563

† These authors contributed equally to this work.

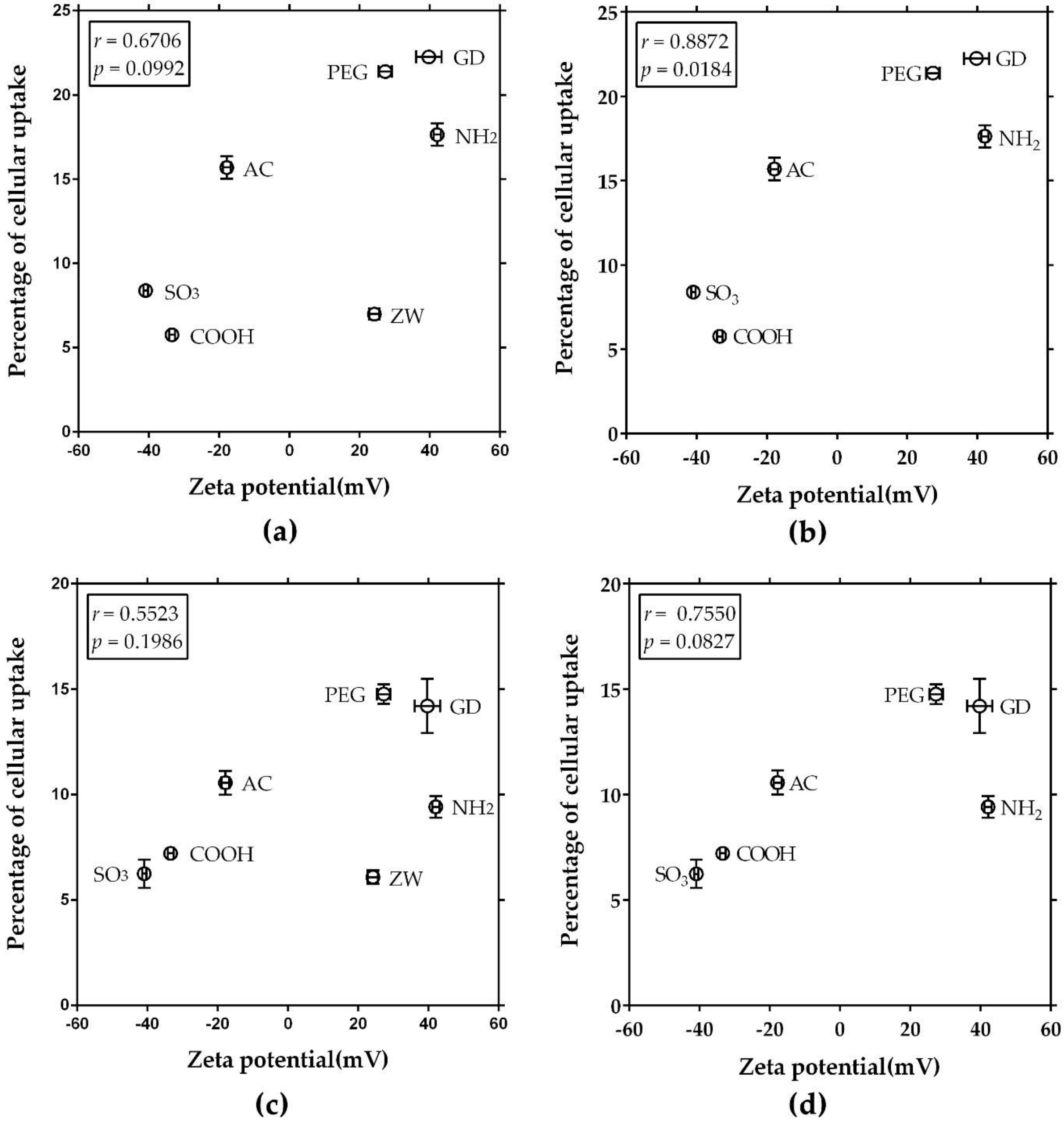

Figure S1. Plotting of values of zeta potential in DW against percentages of cellular uptake. The zeta potential of nanoparticles was plotted against percentages of cellular uptake in THP-1 macrophages using all types of nanoparticles (**a**) or excluding (zw)*F*-PLNP (**b**) which considered as an outlier. The zeta potential of nanoparticles was plotted against percentages of cellular uptake in A549 cells using all types of nanoparticles (**c**) or excluding (zw)*F*-PLNP (**d**) which considered as an outlier. The Pearson's correlation test was applied. Data are presented as mean ± SEM and n = 4.

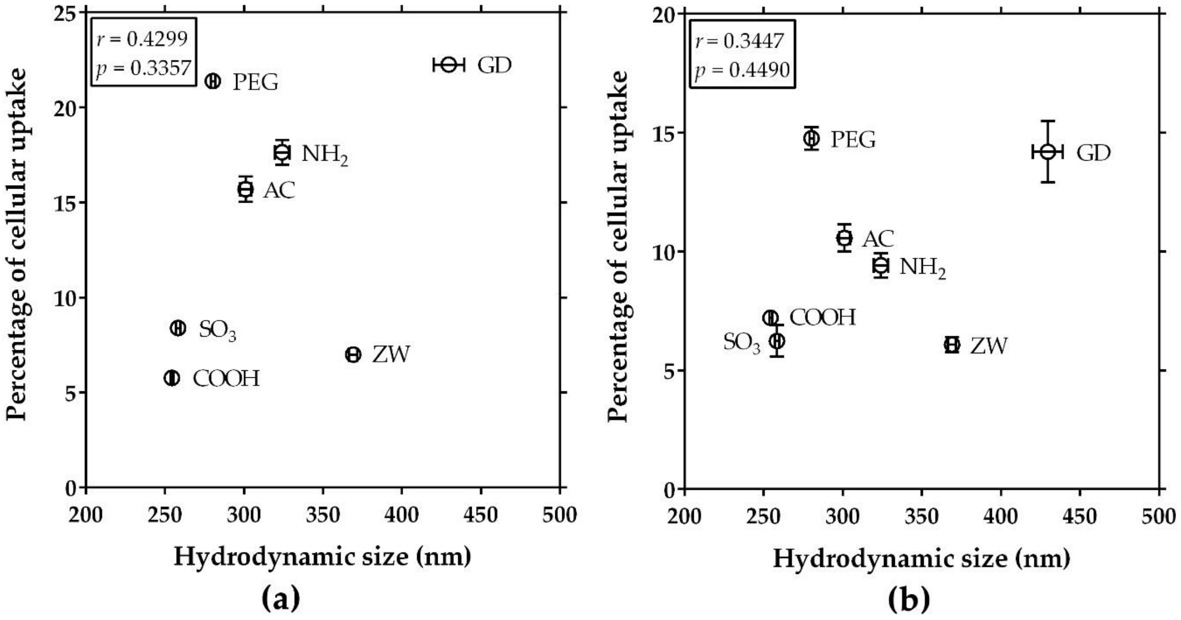

Figure S2. Plotting of values of hydrodynamic size against percentages of cellular uptake. The hydrodynamic size of nanoparticles was plotted against percentages of cellular uptake in THP-1 macrophages (**a**) and A549 cells (**b**). The Pearson's correlation test was applied. Data are presented as mean ± SEM and $n = 4$.
